# Apoptosis Activation in Human Lung Cancer Cell Lines by a Novel Synthetic Peptide Derived from *Conus californicus* Venom

**DOI:** 10.3390/toxins8020038

**Published:** 2016-02-05

**Authors:** Irasema Oroz-Parra, Mario Navarro, Karla E. Cervantes-Luevano, Carolina Álvarez-Delgado, Guy Salvesen, Liliana N. Sanchez-Campos, Alexei F. Licea-Navarro

**Affiliations:** 1 Biomedical Innovation Department, Scientific Research and High Education Center of Ensenada (CICESE), Carretera Ensenada-Tijuana No 3918 Fracc, Zona Playitas Ensenada C.P. 22860, Baja California, Mexico; oroz@cicese.edu.mx (I.O.-P.); karla.cervantes@icgeb.org (K.E.C.-L.); alvarezc@cicese.mx (C.A.-D.); lsanchez@cicese.edu.mx (L.N.S.-C.); 2 Cancer Center, Stanford Burnham Prebys Medical Discovery Institute, 10901 North Torrey Pines Road, La Jolla, CA 92037, USA; mnavarro@sbpdiscovery.org (M.N.); gsalvesen@sbpdiscovery.org (G.S.); 3 International Centre for Genetic Engineering and Biotechnology, Padriciano 99, 34149 Trieste, Italy

**Keywords:** lung cancer, synthetic peptide, apoptotic-related genes, caspase-3 and -7, apoptosis, pathway

## Abstract

Lung cancer is one of the most common types of cancer in men and women and a leading cause of death worldwide resulting in more than one million deaths per year. The venom of marine snails *Conus* contains up to 200 pharmacologically active compounds that target several receptors in the cell membrane. Due to their diversity and specific binding properties, *Conus* toxins hold great potential as source of new drugs against cancer. We analyzed the cytotoxic effect of a 17-amino acid synthetic peptide (s-cal14.1a) that is based on a native toxin (cal14.1a) isolated from the sea snail *Conus californicus*. Cytotoxicity studies in four lung cancer cell lines were complemented with measurement of gene expression of apoptosis-related proteins Bcl-2, BAX and the pro-survival proteins NFκB-1 and COX-2, as well as quantification of caspase activity. Our results showed that H1299 and H1437 cell lines treated with s-call4.1a had decreased cell viability, activated caspases, and reduced expression of the pro-survival protein NFκB-1. To our knowledge, this is the first report describing activation of apoptosis in human lung cancer cell lines by s-cal14.1a and we offer insight into the possible mechanism of action.

## 1. Introduction

Lung cancer is one of the most common types of oncological malignancies and remains the leading cause of cancer-related deaths, representing approximately 15% of all cases worldwide [1,2]. Despite all efforts to advance surgical procedures, radiotherapy and chemotherapy, the five-year survival rate for lung cancer patients has remained almost constant over the past three decades and persists at a dismal 15% [3,4]. Thus, there is an increasing emphasis on strategies to maximize tumor control, prolong survival, minimize chemotherapy side effects and improve quality of life for patients [5]. 

Venoms are an extraordinary source of novel peptides, some of these have found applications to treat several pathologies in humans [6]. The large biodiversity offered by venom peptides, especially conotoxins isolated from the venom of predatory marine snails *Conus*, hold great promise for the development of peptide-based drugs [7]. The genus *Conus* consists of 500–700 species [8,9,10] and the venom of each species contains up to 200 pharmacologically active components that specifically target membrane receptors, ion channels and transporters in the nervous system [11,12,13,14].

Apoptosis, a vital biological process of multicellular organisms is mediated by the activation of proteases named caspases [15,16,17]. Apoptosis and caspase activation may take place via two major signaling systems: (1) the extrinsic or death receptor pathway, which is triggered via specific cell membrane receptors; and (2) the intrinsic or mitochondrial pathway triggered upon disruption of mitochondria and release of cytochrome c [18,19,20,21,22]. A third signaling system has been reported: the granzyme B pathway, where the cytotoxic cell protease granzyme B is delivered to sensitive target cells [23]. Deregulation of apoptosis either by loss of pro-apoptotic signals or by gain of anti-apoptotic signals can lead to initiation, promotion and progression of cancer, and it may also result in therapy failure [24]. Successful elimination of cancer cells from the body depends on activation of cell death by apoptosis, thus developing peptides to stimulate caspase activation and apoptosis execution represent a promising strategy to develop cancer chemotherapeutics [5]. 

In the present study, we analyze the cytotoxic properties of a synthetic peptide derived from the toxin cal14.1a isolated from *Conus californicus*. s-cal14.1a is part of a new superfamily named J_2_ [25] and conserve a cysteine pattern with other *Conus* toxins that are active against acetylcholine nicotinic receptors (nAChRs). The amino acid residues in the sequence of s-cal14.1a and the cysteine pattern are fundamental for the activity and affinity of α-conotoxins [26,27,28]. nAChRs expression was thought to be restricted to neuronal and muscle cells, but emerging research shows that nAChRs are widely expressed in mammalian cells, including caner cells [29]. nAChRs may play key roles in pathogenesis as these interact with its agonists leading to activation of multiple signaling pathways that regulate progression, growth and metastasis of tumors [2,30,31]. Here we studied the cytotoxic properties of s-cal14.1a in four lung cancer cell lines, we quantified the expression of genes involved in execution and regulation of apoptosis namely Bcl-2, BAX and the pro-survival proteins NFκB-1 and COX-2, we also analyzed caspase activity. Our results indicate that s-cal14.1a induces down regulation of anti-apoptosis genes at the same time that leads to activation of DEVD-ase activity that is diagnostic for activity of caspase-3 and -7, two key caspases in the execution of the apoptotic pathway.

## 2. Results

### 2.1. Effect of s-cal14.1a on Lung Cancer Cells Viability 

**Figure 1:**
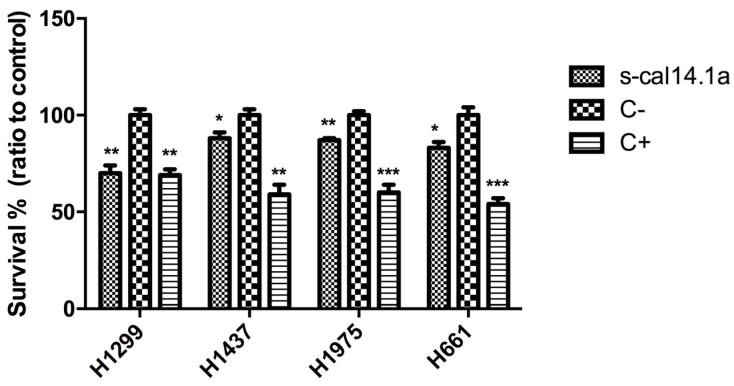
Effect of s-cal14.1a on cell viability. Lung cancer cells H1299, H1437, H1975 and H661 were seeded on 96-well plates and treated with 27 μM of s-cal14.1a for 24 h. Cell viability was determined by measuring absorbance in wells at 490 nm with MTS assay. Results were normalized to untreated cells (C−) to obtain the percentage of cell viability and are expressed as the mean ± SEM. Positive control (C+) 5 μM of staurosporine. Experiments were done in triplicates. * *p* < 0.05, ** *p* < 0.01 and *** *p* < 0.001 *vs.* C− (unpaired *t* Student’s test).

Activity of s-cal14.1a against lung cancer cell lines H1299, H1437, H1975 and H661 was evaluated using the MTS assay [32]; in all cell lines we observed decreased cell viability after 24 h, up to 30%, with respect to untreated cells (Figure 1). In all experiments, staurosporine was used as a positive control (C+). Staurosporine is an alkaloid isolated from *Streptomyces staurosporeus* known to activate apoptosis in several cancer cells [33]. Complete medium was added as negative control (C−).

### 2.2. Expression Analysis of Apoptotic-Related Genes

The effect of s-cal14.1a on mRNA expression of selected genes was analyzed by RT-qPCR. The threshold cycles (CT) of the reference gene (β-actin) and the target genes (Bcl-2, BAX, NFκB-1 and COX-2) were determined in each sample. The relative mRNA expression of each gene analyzed was normalized against β-actin gene and then against the C− of each treatment as calculated by the relative standard curve method [34].

**Figure 2:**
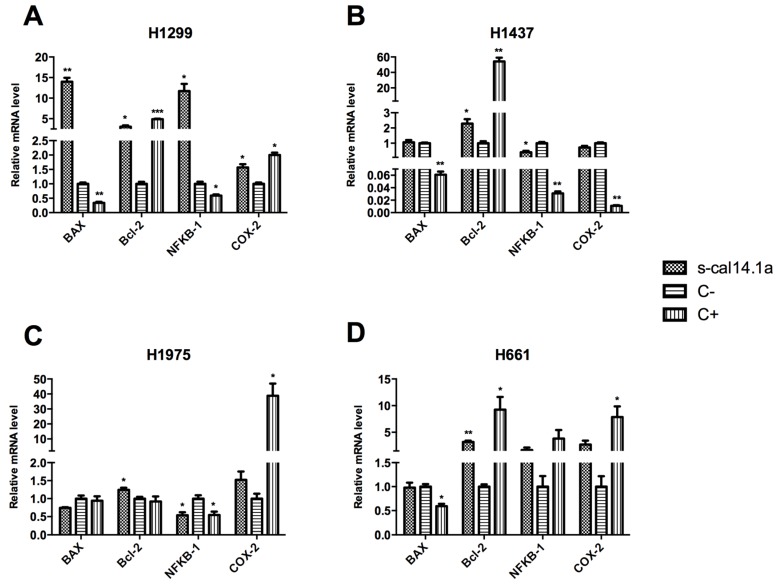
mRNA expression profile of BAX, Bcl-2, NFκB-1 and COX-2 in H1299 (**A**); H1437 (**B**); H1975 (**C**); and H661 (**D**) cell lines. A total of 1 × 10^6^ cells were treated with 54 μM of s-cal14.1a for 24 h. Total RNA was isolated and treated with DNase, 2 μg were reverse-transcribed with SuperScript III Kit, using oligodT_20_ and a random hexamer. mRNA levels were compared by RT-qPCR. Results were normalized to the β-actin gene and expressed as the mean ± SD relative to the negative control (C−, untreated cells). As positive control (C+) cells were treated with 7 μM of staurosporine. Experiments were done in triplicate. * *p* < 0.05, ** *p* < 0.01 and *** *p* < 0.001 *vs.* C− (unpaired Student’s *t*-test).

Cells were incubated with 54 μM s-cal14.1a for 24 h, after this period gene expression was measured. s-cal14.1a increased levels of BAX mRNA in H1299 cells, but C+ decrease its expression (Figure 2A). It is known that BAX codes for a pro-apoptotic protein [35], therefore, we expected similar expression of BAX levels between cells treated with s-cal14.1a and staurosporine. Bcl-2 transcript levels appeared to be vastly increased with s-cal14.1a and the C+, this is contradictory to our expectations because Bcl-2 is an anti-apoptotic protein [36]. Regarding the expression of NFκB-1 and COX-2 in H1299 cell line, cal14.1a induced a level of expression as that observed in the control (Figure 2A).

s-cal14.1a and C+ increased expression of Bcl-2 in H1437 cell line (Figure 2B). NFκB-1 mRNA levels showed a minor decline compared to C+ which had a considerable decrease after 24 h of treatment. BAX and COX-2 genes showed no significant difference compared to C−, but C+ reduced the expression of both genes. Additionally BAX protein levels were analyzed by Western blot. The results obtained (Figure S1A,B) are in agreement with the mRNA expression profile for BAX in H1437 cell line, *i.e.*, treatment with staurosporine decreased the relative protein expression of BAX, while treatment with s-cal14.1a had no effect in protein expression when compared to C−. These findings suggest that treatment with s-cal14.1a induces similar results at the protein and mRNA levels. Taking this into account, we assume that the effect of s-cal14.1a on mRNA expression profile would be similar at the protein level. In H1975 cells treated with s-cal14.1a Bcl-2, mRNA levels were slightly up-regulated (Figure 2C), but the C+ showed no difference compared to C−. BAX expression did not show significant differences in neither s-cal14.1a nor C+ treatments. In H1975 cells, expression levels of NFκB-1 were equivalent in treatment with s-cal14.1a and staurosporine. COX-2 levels showed no difference between non-treated cells and those treated with s-cal14.1a. However, treatment with staurosporine induced considerable increase of COX-2 after 24 h.

Treatment of H661 cells with s-cal14.1a showed a 2-fold increase Bcl-2 levels, while cells treated with staurosporine Bcl-2 expression was increased 8-fold (Figure 2D), as previously shown in the other three cell lines analyzed. Levels of NFκB-1 and COX-2 did not showed significant difference with s-cal14.1a treatment compared to the non-treated control.

Expression of apoptotic-related genes in four cancer cell lines had an interesting outcome. In the case of Bcl-2, its mRNA levels were increased in all the cell lines tested after 24 h of treatment with s-cal14.1a and C+, since Bcl-2 is an anti-apoptotic protein we expected to find decreased levels of this gene. Moreover, expression of the pro-apoptotic gene BAX was increased in H1299 cell (Figure 2A) after the treatment with s-cal14.1a, but in H1437, H1975 and H661 (Figure 2B,C) had no significant difference, both BAX and Bcl-2 genes behaved contrary to expectations. Expression levels of NFκB-1 and COX-2 were different in all cell lines tested, NFκB-1 was increased with s-cal14.1a in H1299 cells, whereas, in H1437 and H1975 were decreased. The NFκB signaling pathway plays an important function in promoting survival and growth of tumor cells [37]. COX-2 was slightly increased with s-cal14.1a and C+ in H1299; in the others cell lines, no significant difference was observed. Although these results are somewhat contradictory to our expectations Bcl-2, BAX, NFκB-1 and COX-2 are only four of the different genes involved in regulation of apoptosis. A more global analysis could reveal changes in gene expression that were not reflected in the genes analyzed. 

### 2.3. Activation of Caspase-3 and -7 in Lung Cancer Cell Lines

To determine if s-cal14.1a promotes cell death in lung cancer cell lines we analyzed apoptosis activation. Apoptosis execution depends on the proteolytic activity of caspase-3 and -7 which in turn cleave proteins substrates that bring cells to metabolic dismay [38]. After incubation of cell lines with s-cal14.1a we assessed caspase-3 and -7 activity by fluorescence microscopy using the commercial kit CellEvent™Caspase-3/7 that is based on the emission of fluorescence upon cleavage of the fluorescently labeled DEVD peptide. In addition, cells were stained with nuclear dyes Hoechst 33342 and propidium iodide (PI), that is also used to identify necrotic or apoptotic cells. After taking images at different time points we calculated the percent of positive cells to caspase-3 and -7 activation and labeled with PI. It is noteworthy that the images do not always match the cell counts in the graphs, this is because the image covers a limited area for all pictures taken and the counting is for all the cells in the field of analysis.

**Figure 3:**
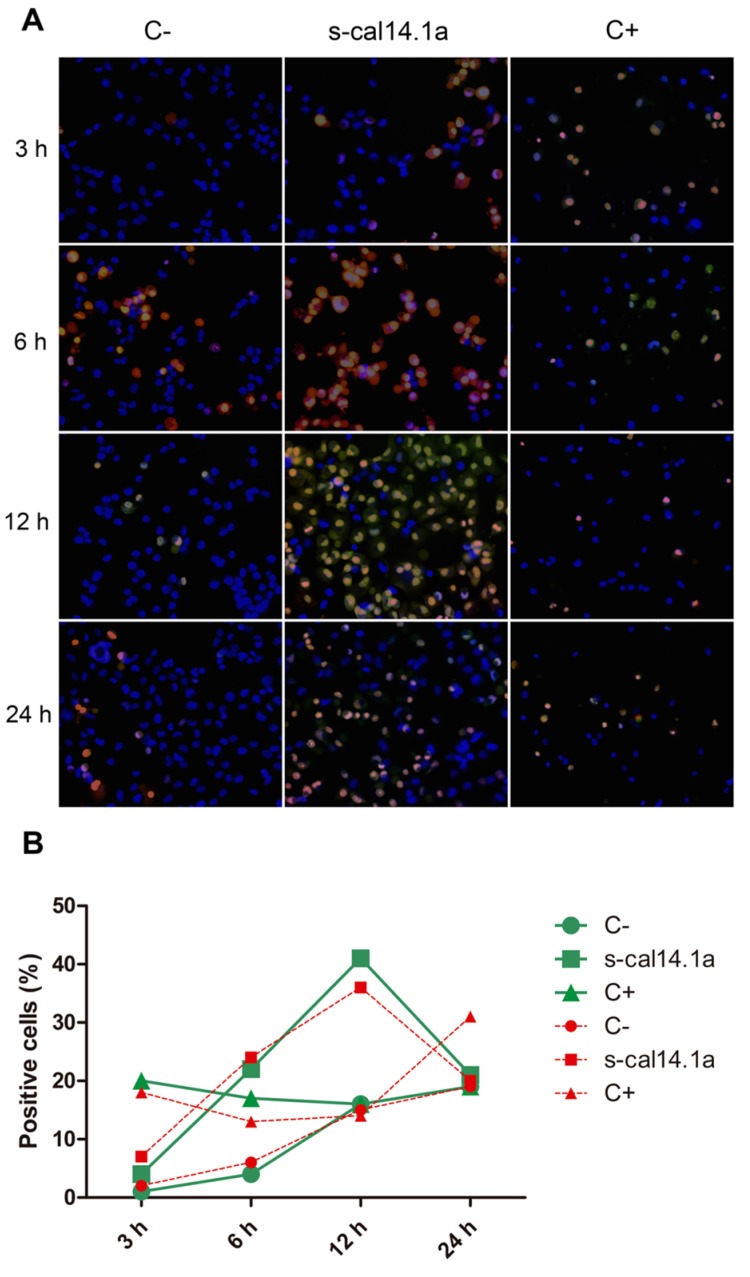
Time course of caspase-3 and -7 activation in cell line H1299. Cells were treated with 27 μM of s-cal14.1a for 3, 6, 12 and 24 h the incubated with CellEvent™Caspase-3/7 (green), Hoechst 33342 (blue) and PI (red) for 30 min at 37 °C and 5% CO_2_. (**A**) Representative images showing untreated cells (C−), cells treated with s-cal14.1a and C+ (1 μM staurosporine) at 460× overall magnification; (**B**) Count of positive cells to the different dyes. Cells stained blue were considered as 100%. Results are expressed as the percentage of cells that are positive to caspase-3 and -7 (green) and PI (red).

After incubation with s-cal14.1a H1299 cells showed major activation of caspase-3 and -7 at 12 h (41% of positive cells Figure 3B) while at 6 h and 24 h the number of positive cells were 22% and 21%, respectively. Cells treated with staurosporine displayed 36% of positive cells throughout the time of the experiment. These results indicate that H1299 cells are more sensitive to s-cal14.1a than to staurosporine.

**Figure 4:**
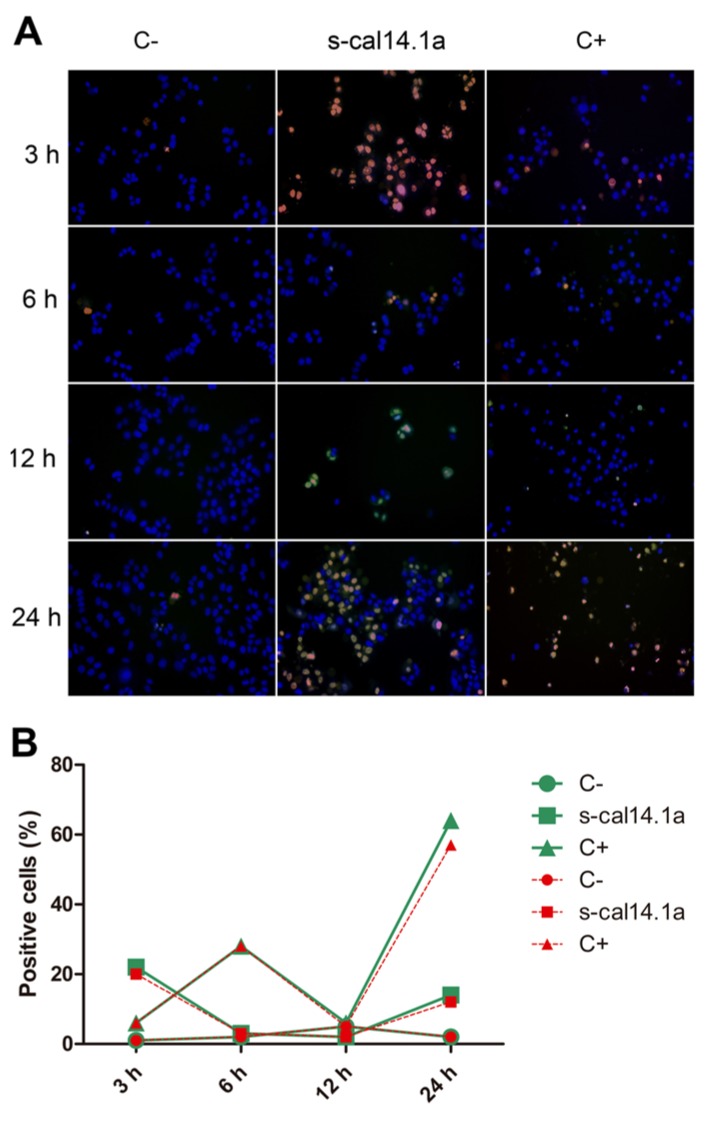
Time course of caspase-3 and -7 activation in H1437 cell line. Cells were treated with 27 μM of s-cal14.1a for 3, 6, 12 and 24 h. (**A**) Representative image showing untreated cells (C−) and cells treated with s-cal14.1a and C+ (staurosporine 1 μM) at 460× overall magnification; (**B**) Graph showing counting positive cells to caspase-3 and -7 activation. Cells stained blue were considered as 100%. Results were expressed as the percentage of cells that are positive to caspase-3 and -7 (green) and PI (red).

Activation of caspase-3 and -7 in the H1437 cell line shows 22% of positive cells after 3 h of treatment with s-cal14.1a (Figure 4B), followed by a considerable decrease at 6 h and 12 h (5%) and a final increase at 24 h (14%). Treatment with staurosporine induced (64%) caspases activation after 24 h of incubation. This result differs from H1299 cells that activate a major percentage of caspases at 6 h and 12 h, which may indicate that activation of caspase-3 and -7 depends on the type of cell and on the stimulus used to induce apoptosis [39]. 

**Figure 5:**
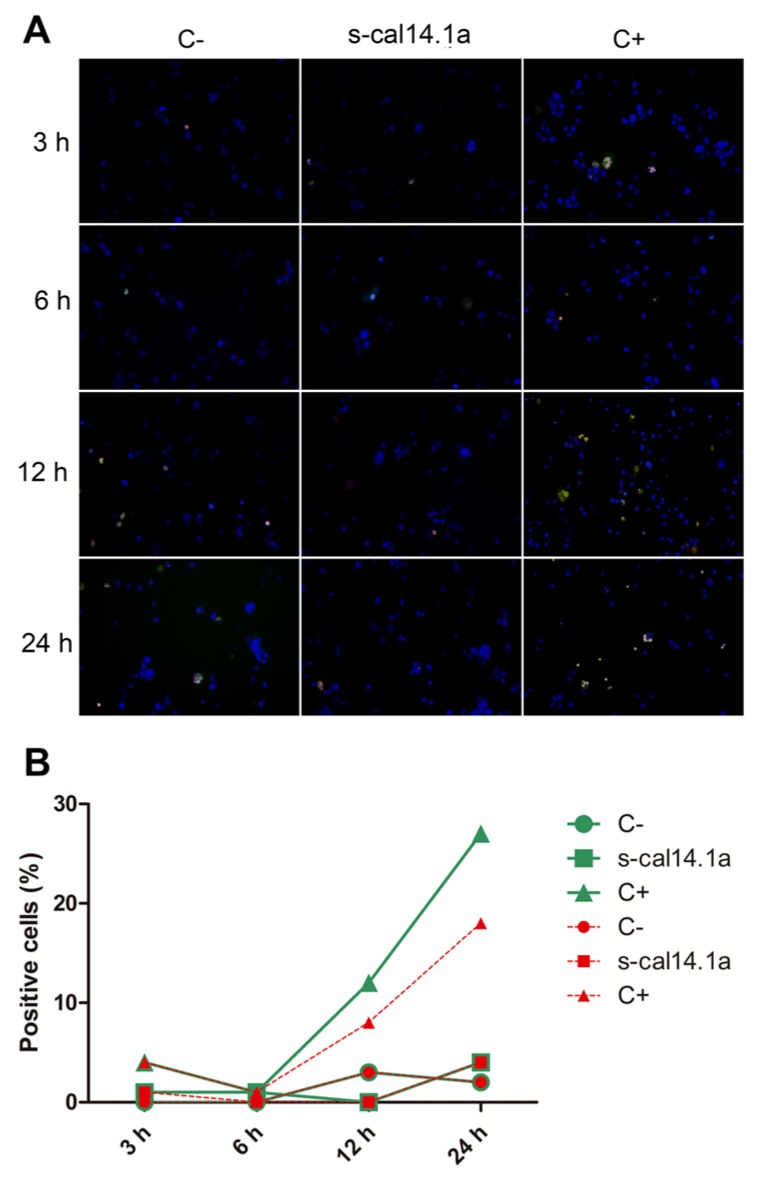
Time course of caspase-3/7 activation in H1975 cell line. (**A**) Representative image showing untreated cells (C−) and cells treated with s-cal14.1a and C+ (1 μM staurosporine) at 460× overall magnification; (**B**) Graph showing counting positive cells to caspase-3 and -7 activation. Cells stained blue were considered as 100%. Results were expressed as the percentage of cells that are positive to caspase-3 and -7 (green) and PI (red).

**Figure 6:**
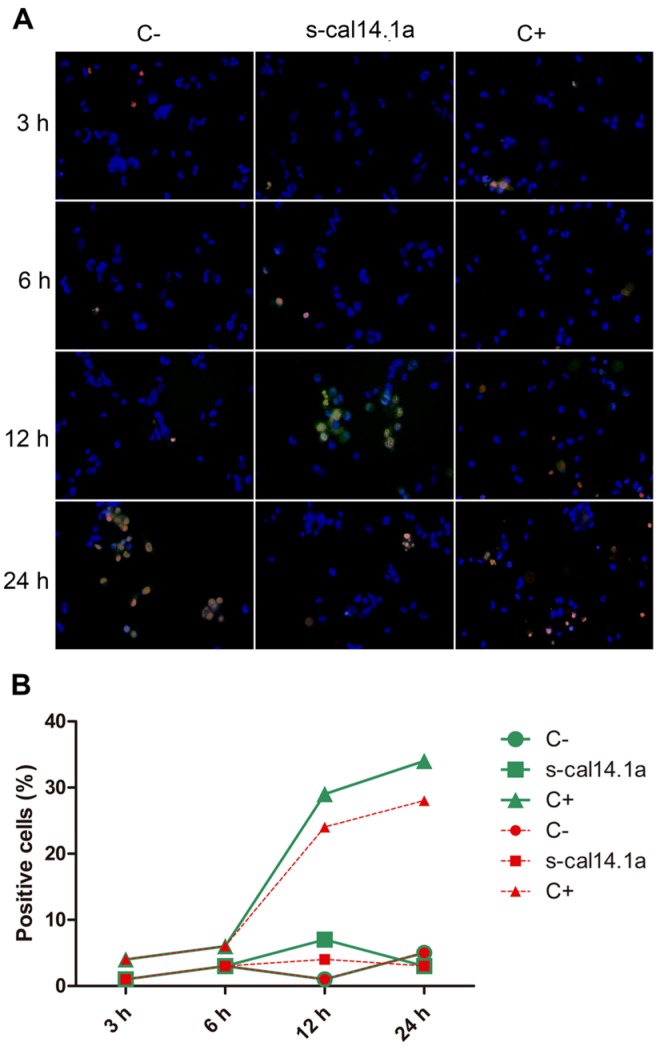
Time course of caspase-3/7 activation in H661 cell line. (**A**) Representative image showing untreated cells (C−) and cells treated with s-cal14.1a and C+ (1 μM staurosporine) at 460× overall magnification; (**B**) Graph showing the number of positive cells to caspase-3 and -7 activation. Cells stained blue were considered as 100%. Results were expressed as the percentage of cells that are positive to caspase-3 and -7 (green) and PI (red).

Regarding H1975 and H661, neither of the two cell lines showed more than 7% of positive cells to caspase-3 and -7 with s-cal14.1a treatment at any time (Figure 5A and Figure 6A). H1975 and H661 cells treated with staurosporine showed 27% and 34% of positive cells, respectively (Figure 5B and Figure 6B). These types of cells appear to be more resistant to apoptosis activation and may need longer incubation with the toxin or a higher concentration.

All cell lines showed a similar percentage of positive cells to PI and caspase-3 and -7. After s-cal14.1a treatment, H1299 and H1437 cells showed a large percent of positive cells to PI. This confirms the results obtained with caspase-3 and -7 and demonstrates that s-cal14.1a induces apoptosis.

### 2.4. Apoptosis-Dependent Cell Death Induced by s-cal14.1a on H1437

These results indicate that s-cal14.1a induces cell death of lung cancer cell lines (up to 30% decrease of survival). To demonstrate that cell death is caused by apoptosis we used small molecule inhibitors of apoptosis and necroptosis. z-VAD-fmk (carboenzoxy-valyl-alanyl-aspartyl-[*O*-methyl]-fluorometylketone) is a cell-permeable pan-caspase inhibitor that irreversibly binds to the catalytic site of these proteases to inhibit activation and execution of apoptosis [40]. To inhibit necroptosis, necrosulfonamide (NSA) was used, a highly specific inhibitor that targets the Mixed Lineage Kinase domain-Like protein (MLKL), a critical component of the necroptotic pathway [41].

**Figure 7:**
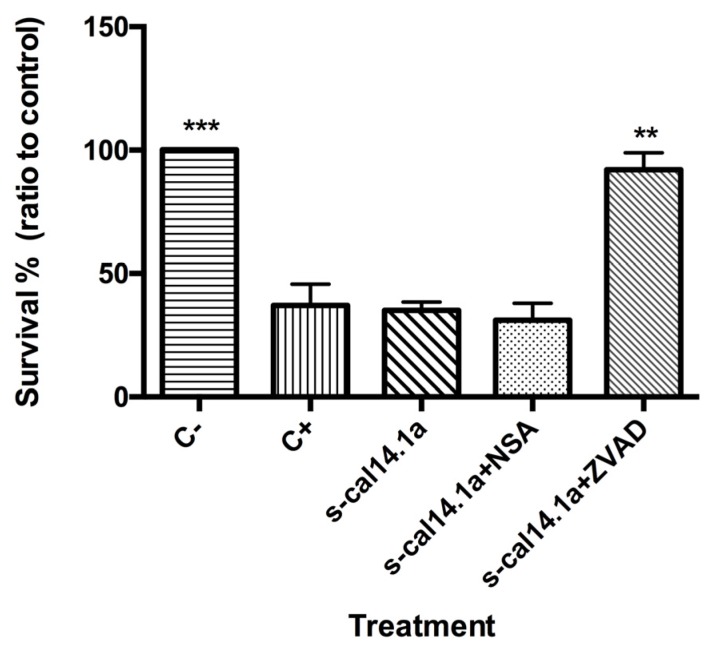
Cell death in H1437 cell line. Cells were seeded on 96-well plates and treated with 27 μM of s-cal14.1a only and s-cal14.1a plus 50 μM NSA (necrosulfonamide) or ZVAD (z-VAD-fmk). Survival was determined with MTS assay. Results were normalized to C− (untreated cells) and are expressed as the mean ± SEM. Positive control (C+) 5 μM of staurosporine. Experiments were done in triplicates. ** *p* < 0.01 and *** *p* < 0.001 *vs.* s-cal14.1a treatment (unpaired *t* Student’s test).

We studied the effect of these two inhibitors on cell death caused by s-cal14.1a in the cell line H1437 was analyzed (Figure 7). We decided to use only H1437 for this assay because its results in apoptosis-related genes expression and capase-3 and -7 activation experiments. H1437 cells were treated with 27 μM s-cal14.1a, 5 μM staurosporine (C+) or preincubated for 1 h with 50 μM NSA or (Z-VAD) then treated with s-cal14.1a. After 24 h of incubation, viability was determined with MTS assay.

The result shows that s-cal14.1a decreased the survival rate to less than 50% and the same was observed for the C+. Cells preincubated with NSA and s-cal14.a survived to the same extent as those treated with s-cal14.1a only. NSA fails to inhibit cell death suggesting that cell death induced by s-cal14.1a is a necroptosis-independent event. On the contrary, cells pretreated with Z-VAD prior to s-cal14.1a addition survived as much as the non-treated cells.

## 3. Discussion

### 3.1. Cytotoxic Effect of s-cal14.1a on Four Lung Cancer Cell Lines

α-conotoxins are small peptides (12–20 amino acid residues) isolated form the venom of cone snails and are known to target muscle- and neuronal-type nAChRs. α-conotoxins are among the smallest conopeptides isolated from *Conus sp.* These toxins bind at the acetylcholine binding site in the nAChR acting as competitive antagonists [42]. In this work we analyzed the effect of s-cal14.1a on cell viability, this synthetic peptide has been characterized to target nAChRs [25]. The amino acid sequence and the cysteine pattern in s-cal14.1a are highly conserved in other *Conus* toxins that are active in nAChR, like the peptide It14a from *Conus litteratus* [43]. In lung cells nAChRs act as central mediators in the activation of pathways that promote growth, progression, and metastasis of tumors. nAChRs also regulate a network of stimulatory and inhibitory neurotransmitters that govern the synthesis and release of growth, angiogenic, and neurogenic factors in the microenvironment of cancer cells and in distant organs [2,29]. Therefore, blocking signaling cascades downstream of nAChRs with antagonists may result in cell death [1]. Expression of nAChRs subunits such as α2, α3, α4, α5, α6, α7, α9, β2, and β4 has been detected in lung cancer while overexpression is directly related to lung cancer pathogenesis and resistance [2,3,44,45]. We determined the mRNA levels of expression of nAChR subunits in a panel of lung cancer cell lines, H1299, H1437, H1975 and H661 (Figure S2A–D). All cell lines tested expressed α5; H1299 (Figure S2A) and H1437 (Figure S2B) also express minor levels of α7. H661 expresses α3, α7 and α9 as well (Figure S2D). Since the lung cancer cells studied express nAChR subunits we hypothesize that s-cal14.1a may target nAChRs and block signaling downstream.

By treating different cancer cell lines with s-cal14.1a we discovered a cytotoxic effect. Some of the cell lines showed a reduction in cell viability of up to 40% (Figure 1); we speculate that s-cal14.1a may target nAChRs thus blocking the signaling cascade, resulting in cell death. Signaling through nAChRs begins with binding of agonists such as ACh (acetylcholine), nicotine and tobacco nitrosamines, causing a conformational change that leads to channel opening and influx of Na^+^ and Ca^2+^, and efflux of K^+^. Resulting membrane depolarization opens voltage-operated calcium channels, leading to additional flow of Ca^2+^. Ca^2+^ influx triggers secretion of mitogenic factors and activates signaling cascades involved in cell proliferation, apoptotic inhibition, migration and angiogenesis [1,29,46]. Blocking nAChR channels with an antagonist such as s-cal14.1a could prevent downstream signaling resulting in inhibition of, proliferation, migration, and angiogenesis, and apoptosis. A study focusing on antagonists that target nAChRs as a therapeutic alternative to treat cancer has provided significant insights into their mechanisms of action [29]. Among these nAChRs, the α7-nAChR subunits are known to be overexpressed in lung cancer; *in vitro* experiments suggest that malignant growth can be halted using α-conotoxins, which are competitive antagonists of nAChRs [47]. Alternatively, the exquisite specificity of toxins has been used to target receptors for drug delivery purposes. The α-conotoxin ImI was used as a targeting peptide to efficiently deliver the chemotherapeutic agent paclitaxel to the α7-nAChR to tumor cells in breast cancer [48]. nAChRs are believed to play an important role in nicotine-related lung cancer pathogenesis [49]. Although nicotine itself is not a carcinogen, it is the principal reinforcing component in cigarette smoke and can promote tumor growth by inducing proliferation, angiogenesis, migration and invasion [50].

A previous study demonstrated that exposure to nicotine and its derivatives, which bind to nAChRs on bronchial epithelial cells, can regulate proliferation and apoptosis of non-small cell lung cancer (NSCLC) by activating the Akt pathway [51,52]. In particular, α5-nAChR has been found to be closely associated with lung cancer risk and nicotine dependence. It has been reported that nicotine interacts with α5-nAChR on the surface of A549 cells leading to activation the ERK and Akt signaling, upregulation of VEGF levels and HIF-1α signaling. Ultimately, this promotes cell proliferation, angiogenesis, and invasion [2]. nAChRs have also been noted to promote a nicotine-stimulated epithelial-to-mesenchymal transition (EMT) in a variety of human cancer cell lines, especially in small cell lung cancer and non-small cell lung cancer [53].

It has been demonstrated that α9-nAChR is a key molecule in mediating the nicotine-enhanced migration of breast cancer cells through regulation of EMT markers, while pretreatment with a nAChR inhibitors abolishes these effects [47,54]. nAChRs are important therapeutic targets, and development of new antagonists that specifically block these targets are potentials candidates for alternative cancer therapy.

Based on the above studies, we propose that s-cal14.1a may induce cell death by acting on the nAChRs expressed in lung cancer cells and may be an alternative approach to design novel therapeutic compounds. Synthetic peptides based on native sequence of conotoxins can be also used as tools for nAChR characterization.

### 3.2. Apoptosis-Related Genes as Regulators of Cell Death

Apoptosis is a process by which damaged, unattached, mutant and aged cells are eliminated, aberrations in this pathway can lead to a variety of diseases including cancer [55,56]. Targeting components of the apoptotic pathway as a therapeutic approach in cancer is supported by the finding that aberrant apoptosis is central to tumor growth and progression. Indeed, suppression of apoptosis is a recognized hallmark of cancer [57]. Novel approaches to activate the apoptotic pathway may result in induction of death in cancerous cells. Overexpression of anti-apoptotic Bcl-2 family members is known to cause apoptosis or therapy resistance in a wide range of tumors. Development of therapies that target these modulators of apoptosis appears to be a promising approach [58,59].

NFκB family is a central mediator of immune, inflammatory, stress responses, apoptosis and cell proliferation. NFκB is constitutively activated in a variety of leukemia and solid tumors, including lung cancer and plays a critical role in promoting the survival and growth of tumor cells [37,60,61]. COX-2 is an inducible form of cyclooxygenase that represents a potential pharmacologic target to prevent and treat a variety of malignancies [62]. Overexpression of COX-2 has been shown in various cancer types and has been linked with shorter survival of patients with lung caner [63].

We evaluated the role of a synthetic peptide s-cal14.1a, in human lung cancer cell lines*.* s-cal14.1a had a cytotoxic effect on four different cell lines, decreasing the cell viability by 40%. In order to elucidate how some genes involved in apoptosis regulation are expressed in H1299, H1437, H1975 and H661 lung cancer cells after treatment with s-cal14.1a, the expression of apoptotic-related genes were determined and the induction of caspase-3 and -7 activation was analyzed.

This study demonstrates that s-cal14.1a induces cell death of lung cancer cell lines, gene expression levels of proteins involved in regulating and executing apoptosis reveal an interesting pattern for Bcl-2 and BAX mRNA levels (Figure 2A–D). In all cell lines we detected down-regulation of BAX and up-regulation of Bcl-2, except in H1299 that showed 15-fold increase of BAX after treatment with s-cal14.1a. It has been reported that in human gastric adenocarcinoma BAX expression is significantly down-regulated during the first 24 h of treatment with etoposide, cisplatin or taxol, however levels gradually increased in the next 48 and 72 h [17]. Another work showed that the antineoplastic drug 5-FU decreased the mRNA levels of Bcl-2 in the first 48 h, which coincided with the up-regulation of BAX, after 72 h of treatment Bcl-2 was up-regulated and the levels of BAX were decreased in human gastric adenocarcinoma [64]. Extended time lapse-treatment and dose-dependent analysis of cells treated with s-cal14.1a may reveal a different expression pattern for Bcl-2 and BAX. The data presented here may be the result of a cytotoxic effect independent of Bcl-2 and BAX.

Expression of NFκB-1 in H1299 cells (Figure 2A) was considerably increased after treatment with s-cal14.1a. H1437 and H1975 cells (Figure 2B,C) showed down-regulation of NFκB-1 after the treatment with s-cal14.1a. In the three cell lines, staurosporine, an activator of apoptosis, showed the same expression pattern observed in cells treated with s-cal14.1a. Expression of COX-2 (Figure 2D) does not reveal significant differences. These results indicate that most likely s-cal14.1a promotes cell death via an NFκB-1-dependent pathway. Under certain conditions, NFκB can promote or amplify cell death, however most of these effects relate to the extrinsic pathway, including expression of genes that code for the death receptor Fas or its ligand FasL [61]. Almeida *et al.* (2014) report that cisplatin-resistant cells have active NFκB signaling and inhibition of NFκB translocation to the nucleus enhances apoptosis in head and neck squamous cell carcinoma [65]. Targeting NFκB is a potential therapeutic approach to overcome chemoresistance and radioresistance for cancer treatment [66].

### 3.3. Activation of Caspase -3 and -7 and Apoptosis by s-cal14.1a

Caspases are a family of proteins that maintain homeostasis by regulating cell death and inflammation, they perform these functions by means of limited proteolytic cleavage in specific aspartate residue of their substrates [67,68]. The caspase gene family can be divided into two functional subgroups based on their roles; inflammatory caspases (caspase-1, -4, and -5) regulate cytokine maturation and inflammatory responses. Apoptotic caspases are further divided into two functional subgroups, initiator of apoptosis caspases (caspase-2, -8, -9, and -10) and effector caspases (caspase-3, -6, and -7) [69,70,71]. In this work, activation of caspase-3 and -7 induced by s-cal14.1a was analyzed using the commercial kit CellEvent™Caspase-3/7 Green Detection Reagent. Treatment of H1299, H1437, H1974 and H661 cancer cell lines was performed with s-cal14.1a for 3, 6, 12 and 24 h. H1299 cells treated with cal14.a showed caspase -3 and -7 activation in up to 40% of cells analyzed at 12 h of incubation (Figure 3A,B), staurosporine induced caspase activation only in 20% of the cells at 24 h. Cell line H1437 (Figure 4A,B) underwent considerable activation of caspase-3 and -7 (20%) at 3 h and 24 h of incubation, while treatment with staurosporine showed caspase activation in 60% of the cells after 24 h. These results indicate that maximum activation of caspase-3 and -7 is reached between 6 and 12 h of treatment. It is known that the basic mitochondrial pathway of apoptosis, from the initial trigger to destruction of the cell, can take hours or even days. However, the events that lead to caspase activation often take about ten minutes. This can occur at any time following the induction of apoptosis by a death signal [72]. Nevertheless, in HL60 cells incubation with TNF-α/CHX (tumor necrosis factor-α and cycloheximide), 50% of the cells undergo apoptosis during the initial 6 h. In HUVECs cells the same treatment leads to apoptotic death during the first 18 h of incubation [39]. The response of H1975 and H661 cells treated with s-cal14.1a (Figure 5A,B and Figure 6A,B) had no relevant outcome, neither of cell lines showed a significant caspase activation. H1975 and H661 cells treated with staurosporine had 30% and 40% of positive cells, respectively. H1975 and H661 cells may be insensitive to s-cal14.1a or may need higher doses of s-cal14.1a or extended incubation.

Caspase-3 is the main executioner of apoptosis and can be activated through both extrinsic and intrinsic signaling pathway [23]. There is evidence that loss of expression or function of caspase-3 can render breast cancer cells resistant to apoptosis. These findings may have important clinical implications, not only as disease marker, but also as therapeutic target for different types of cancer [73]. This may explain the lack of caspase activity in untreated lung cancer cells (C−). They showed less than 20% of positive cells (Figure 3, Figure 4, Figure 5 and Figure 6). We demonstrate that caspase activity was increased after treatment with s-cal14.1a. Thus, s-cal14.1a could be considered as potential apoptosis activator in lung cancer. Caspase-3 has been shown to interfere with NFκB activation, in this case, caspase-3 cleaves IκBα generating a cleavage fragment that potentially acts as a constitutive inhibitor of NFκB [74]. It was also reported, that proteolysis by caspase inactivate NFκB proteins and transform them into dominant negative factors, still capable of DNA binding but without its transactivation potential [61]. This may explain the reduced expression levels of NFκB-1 and the activation of caspase-3 and -7 in H1437 after the treatment with s-cal14.1a. The fact that s-cal14.1a plus ZVAD cause an inhibition of cell death, support our previous findings that s-cal14.1a induces apoptosis, a process dependent of caspase activity. These data provide us a promising pathway for s-cal14.1a on H1437 cell-death. However, further research is needed to explain details the mechanism of action of s-cal14.1a.

## 4. Materials and Methods

### 4.1. Cancer Cell Lines

Human non-small cell lung cancer H1299 (carcinoma), H1437 (adenocarcinoma), H1975 (adenocarcinoma) and H661 (large cell carcinoma) were cultured at 37 °C with 5% CO_2_ in RMPI-1640 supplemented with 10% *v*/*v* fetal bovine serum (FBS heat inactivated from Sigma-Aldrich, St. Louis, MO, USA) and 1% antibiotic-antimycotic (10,000 units penicilin, 10 mg sterptomycin, and 25 mg/mL amphotericin B per mL, Sigma-Aldrich). All cell lines were acquired from IECSA (México D.F., México).

### 4.2. Cell Viability Assay

Cell viability was determined using the CellTiter 96 Aqueous One Solution Cell Proliferation Assay Kit MTS (Promega, Madison, WI, USA). The MTS assay is based on the conversion of a tetrazolium salt into a colored, aqueous soluble formazan product by dehydrogenase enzymes in metabolically active cells. The amount of formazan produced is directly proportional to the number of living cells in culture. Two hundred fifty thousand cells per two hundred milliliter were seeded in triplicate in 96-well plates and were treated with cal14.1a (27 μM) and C+ (5 μM) for 24 h. Twenty microliters (3-(4,5-dimethylthiazol-2-yl)-5-(3-carboxymethoxyphenyl)-2-(4-sulfophenyl)-2H-tetrazolium, inner salt (MTS)) were added, and the amount of formazan converted by viable cells was determined by measuring absorbance at 490 nm on a 96-well microplate reader EPOCH (BioTek, Winooski, VT, USA). Results are expressed as the percentage of treated to untreated cells.

### 4.3. RNA Extraction and Quantitative Real-Time PCR (RT-qPCR) Analysis

Total RNA was isolated using Tri Reagent^®^ (Sigma-Aldrich) and quantified by NanoDrop™ Lite Spectrophotometer (Thermo Scientific, Waltham, MA, USA). Two micrograms of RNA were reverse transcribed using a mix of oligodT_20_/random hexamers in the Superscript III cDNA synthesis kit (Invitrogen, Waltham, MA, USA), according to manufacturer’s instructions. Real time PCR reactions were performed using SybrGreen master mix in a 7500 real time PCR system (Applied Biosystems, Waltham, MA, USA). Primer sets specifics for the mature coding sequence of each gene were designed using BLAST (NIH, Bethesda, MD, USA). Forward and reverse primers were designed from the *mRNA sequence* (*GenBank Accession* enlisted in Table 1). To calculate the changes in mRNA expression, we first normalized mRNA expression of target gene to the β-actin mRNA expression in a given sample; the mRNA expression for each gene in the experimental treatment was compared with the level of mRNA expression in the non-treated cells. Fold changes in the mRNA expression are expressed as the mean ± SD relative to the negative control (untreated cells).

**Table 1:** Primers sequences and GenBank accession number used for RT-qPCR.

Gene	Primer	*Genbank Accession* Number and Reference
β-actin	F: GCGAGAAGATGACCCAGATC R: CCAGTGGTACGGCCAGAGG	BRWS1
Bcl-2	F: ATGTGTGTGGAGAGCGTCACC R: TGAGCAGAGTCTTCAGAGACAGCC	BC027258.1
BAX	F: TGGCAGCTGACATGTTTTCTGAC R: TCACCCAACCACCCRGGTCTT	NM_004324.3
NFκB-1	F: CGCCGCTTAGGAGGGAGA R: AGGTATGGGCCATCTGCTGT	NM_003998.3
COX-2	F: TGCATTCTTTGCCCCAGCACT R: AAAGGCGCAGTTTACGCTGT	Inoue *et al.*, 2013 [75]

### 4.4. Caspase-3 and -7 Activity Assay

Intracellular caspase-3 and-7 activity was quantified by fluorescence microscopy using the CellEvent™Caspase-3/7 Green Detection Reagent (Life Technologies, Waltham, MA, USA). Cells were seeded in 96-well plates (Corning) after 3, 6, 12 and 24 h of cal14.1a (27 μM) and C+ (staurosporine, 1 μM) treatment. Plates were treated with 5 μM of CellEvent™Caspase-3/7 Green Detection Reagent (Life Technologies) this kit relies on the cleavage of the tetrapeptide substrate DEVD conjugated to a quenched fluorophore. 10 μg/mL of Hoechst 33342 (Life technologies) and 50 μg/mL of propidium iodide (PI) (Sigma-Aldrich) were used as reference for nucleus staining. The plates were incubated for 30 m at 37 °C with 5% CO_2_ and exposed to corresponding filter (blue channel: 390-40/446-33 nm, green channel: 482-18/532-59 nm and red channel: 586-15/646-68 nm) on the fluorescence microscope.

### 4.5. Cell Imaging

Cell images were obtained with an inverted microscope EVOS FLoid Cell Imaging Station (Life technologies) at 20× magnification. Each image was edited on a computer using ImageJ. 

### 4.6. Statistic Analysis

All data were analyzed under GraphPad Prism5 software (La Jolla, CA, USA). Student *t* test was applied for comparing differences among treatments. A *p* value ≤ 0.05 was considerate statistically significant.

## 5. Conclusions

To the best of our knowledge, this study demonstrates that a novel synthetic peptide s-cal14.1a induces apoptosis via caspase-3 and -7 activation in lung cancer cell lines. This information elucidates an alternative study and treatment for lung cancer cells. The possibility to develop agents that directly target apoptotic mechanisms has generated excitement and could lead to more effective therapies with less toxic side effects.

## Supplementary Materials

The following are available online at www.mdpi.com/2072-6651/8/2/38/s1.

## Abbreviations

The following abbreviations are used in this manuscript:

nAChRs: acetylcholine nicotinic receptors
ACh: acetylcholine
PI: propidium iodide
NSCLC: non-small cell lung cancer
EMT: epithelial-to-mesenchymal transition
TNF-α/CHX: tumor necrosis factor- α and cycloheximide
