# Supplementary material for: Apoptosis Activation in Human Lung Cancer Cell Lines by a Novel Synthetic Peptide Derived from Conus californicus Venom
Source: Toxins (Basel). 2016 Feb 5;8(2):38. doi: 10.3390/toxins8020038 (PMC4773791; doi:10.3390/toxins8020038)
Supplement: Supplementary file 1 [file toxins-08-00038-s001.pdf]

Toxins **2016**, *8*, 38; doi:10.3390/toxins8020038

Supplementary Materials: Apoptosis Activation in Human Lung Cancer Cell Lines by a Novel Synthetic Peptide Derived from *Conus californicus* Venom

Irasema Oroz-Parra, Mario Navarro, Karla E. Cervantes-Luevano, Carolina Álvarez-Delgado, Guy Salvesen, Liliana N. Sanchez-Campos and Alexei F. Licea-Navarro

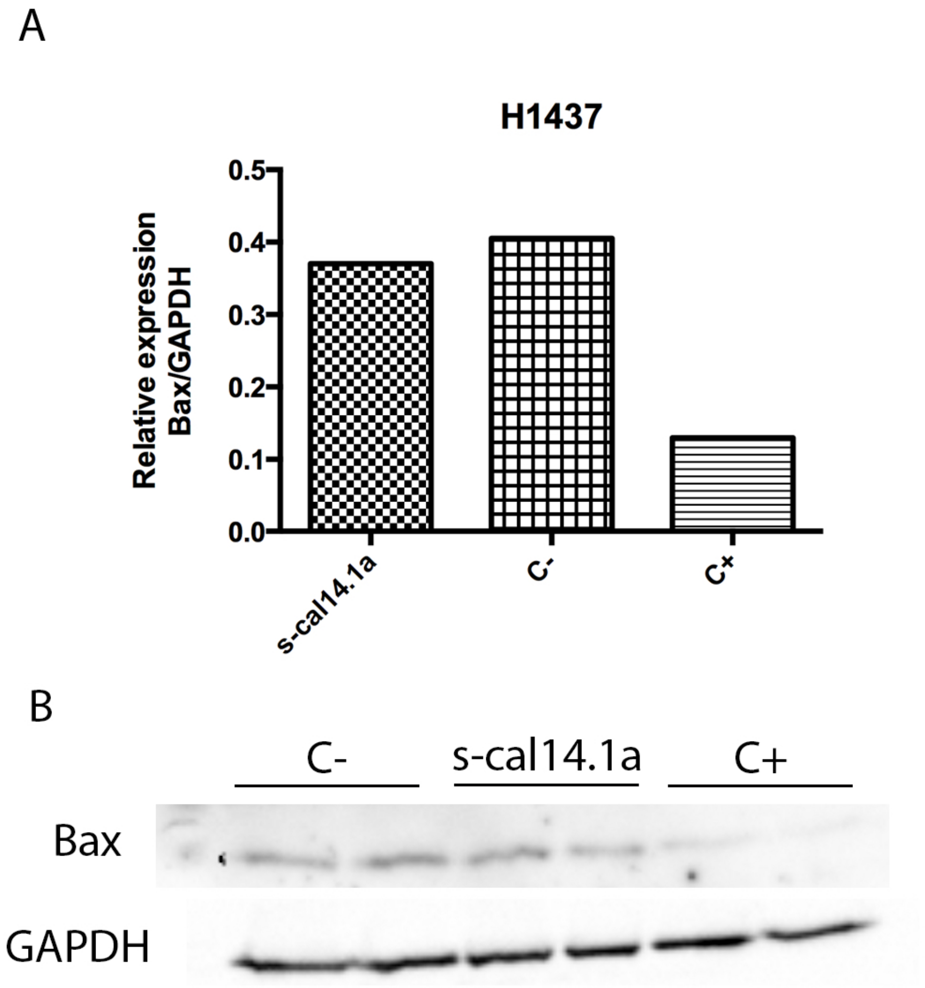

Figure S1. BAX protein levels expression. H1437 cells were seeded and incubated at standard culture conditions for 24 h. Cells were either treated for 24 h with 27 μM of the synthetic peptide s-cal14.1a, 1 μM staurosporine (C+) or left untreated (C−). Total protein was extracted and separated by SDS-PAGE. Protein expression was analyzed by Western blot, using monoclonal antibodies against Bax (sc-7480) and GAPDH (sc-sc-365062). GAPDH was used as a loading control and the results are shown as the relative expression of Bax/GAPDH and are representative of two experiments. (**A**) Densitometric analysis of Bax protein expression. (**B**) Western blots for Bax (upper panel) and GAPDH (lower panel).

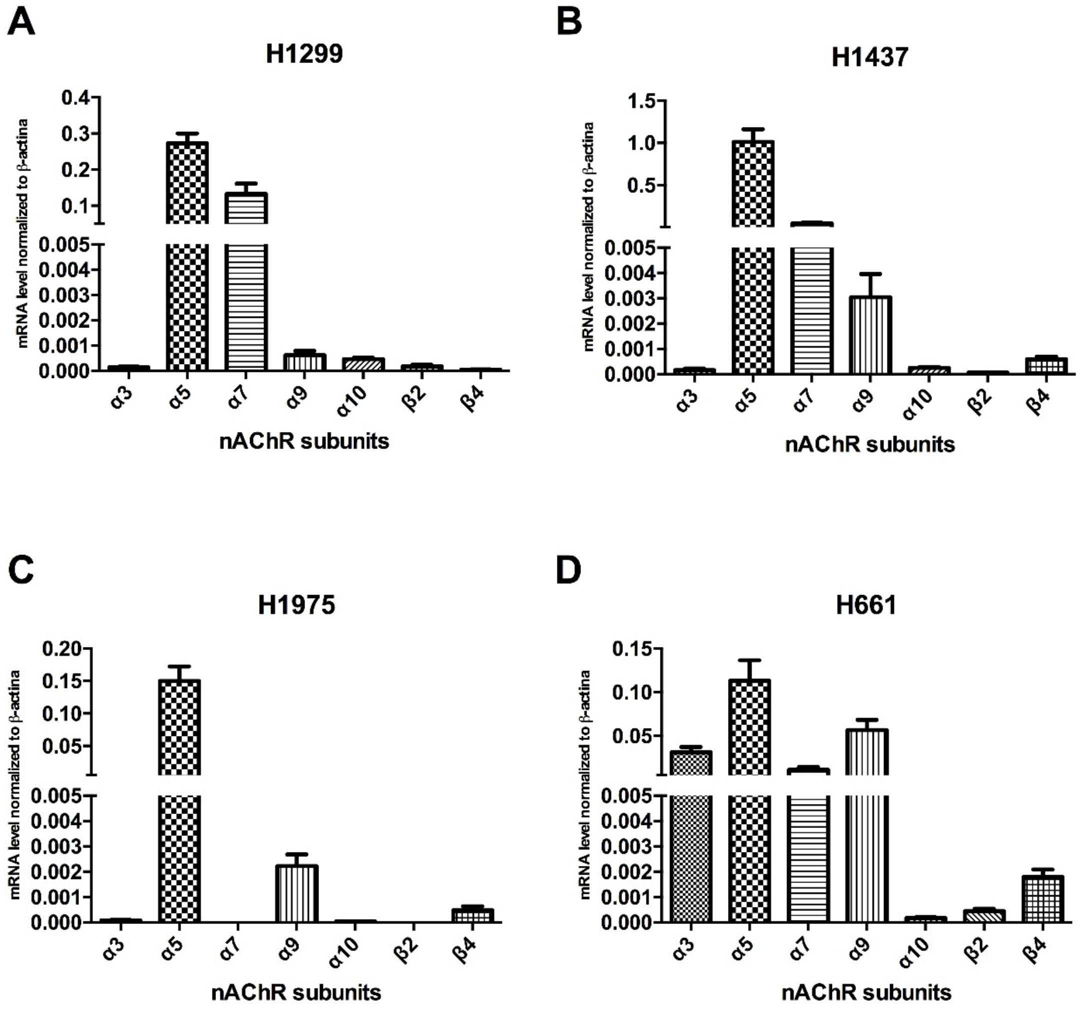

Figure S2. mRNA expression of nAChR subunits in H1299 (**A**), H1437 (**B**), H1975 (**C**) and H661 (**D**) cell lines. Total RNA was isolated and treated with DNase, 2 μg were reverse-transcribed with SuperScript III Kit, using oligodT_{20} and a random hexamer. mRNA levels were compared by RT-qPCR. Results were normalized to the β-actin gene and expressed as the mean ± SD.
